# Evaluation of School-Age Children’s Intelligence Quotient and Their Chronic Exposure to Trace Elements in Ambient Air

**DOI:** 10.7759/cureus.37532

**Published:** 2023-04-13

**Authors:** Heba M Adly, Abdullah A Saati, Abdullah A Khafagy, Maher N Alandiyjany, Saleh A. K Saleh

**Affiliations:** 1 Department of Community Medicine and Pilgrims Health Care, Faculty of Medicine, Umm Al-Qura University, Makkah, SAU; 2 Department of Laboratory Medicine, Faculty of Applied Medical Sciences, Umm Al-Qura University, Makkah, SAU; 3 Quality and Development Affairs, Batterjee Medical College, Jeddah, SAU; 4 Department of Biochemistry, Faculty of Medicine, Umm Al-Qura University, Makkah, SAU; 5 Oncology Diagnostic Unit, Faculty of Medicine, Ain Shams University, Cairo, EGY

**Keywords:** public health program development, iq, children, exposure, trace elements

## Abstract

Background

Children’s exposure to different trace elements in their air, water, and food or even present in paints or toys can affect their intelligence quotient (IQ) score. However, this correlation needs to be analyzed and evaluated in different contexts. This study aimed to investigate the associations between airborne concentrations of lead (Pb), manganese (Mn), cadmium (Cd), chromium (Cr), and arsenic (As) and intellectual function in school-age children in Makkah, Kingdom of Saudi Arabia.

Methodology

Our cohort study aimed to explore the link between exposure to various trace elements in the surrounding air and the IQ scores of children residing in the vicinity of Makkah. We included 430 children in the study and collected information about demographic and lifestyle factors using a structured questionnaire. We employed a mini volume sampler (MiniVol, AirMetrics, Springfield, OR, USA) to collect 24-hour PM10 samples from five locations in Makkah, representing various residential areas with small-to-medium industrial activities and traffic load. We analyzed the samples for Pb, Mn, Cd, Cr, and As concentrations using inductively coupled plasma-mass spectrometry with Perkin Elmer 7300 (Perkin Elmer, Waltham, MA, USA). The combined impact of heavy metals on continuous outcomes was assessed using the Bayesian kernel machine regression model.

Results

The mean atmospheric concentrations of Pb, Mn, Cd, Cr, and As in summer were 0.093, 0.006, 0.36, 0.15, and 0.017 µg/m^3^, respectively, while in winter, they were 0.004, 0.003, 0.12, 0.006, and 0.01 µg/m^3^, respectively. The findings of our study revealed that children's IQ scores were independently associated with co-exposure to the five metals, namely, Pb, Mn, Cd, Cr, and As.

Conclusions

This study demonstrates a link between combined exposure to five heavy metals (Pb, Mn, Cd, Cr, and As) and children’s IQ scores. Regularly evaluating trace elements in children’s biological samples is crucial to comprehend their effects on cognitive growth. To explore the possible future health risks of multimetal exposures and their interaction effects, it is imperative to conduct additional studies that involve repeated biological measurements of metal concentrations.

## Introduction

Exposure to trace elements can adversely affect human health, with the developing brain being particularly susceptible [1]. Various trace elements can accumulate in the human body over time and are easily detectable in biological samples. Observational clinical studies have shown harmful effects from early developmental exposures [2]. Numerous studies have suggested that environmental trace elements can impact the development of intelligence quotient (IQ) in children [3-5]. Some of these environmental heavy metals, such as lead (Pb), cadmium (Cd), manganese (Mn), tin (Sn), antimony (Sb), arsenic (As), and titanium (Ti), may contribute to reduced IQ levels [6-8].

Pb exposure has been linked to neurodevelopmental deficits associated with decreased IQ scores [9] and declines in school performance [10], resulting in significant economic losses to society [11,12]. Another neurotoxic trace element is As. Studies on teenagers who experienced poisoning as infants revealed lower IQ scores and an increased prevalence of central nervous system disorders, including developmental disabilities and epilepsy [13]. Arsenic neurotoxicity’s long-term neurobehavioral and developmental effects remain unclear [14]. Evidence suggests that children with increased exposure to As exhibit neurodevelopmental deficits, including those exposed to metallurgical industries [15]. However, verifying long-term As exposure is challenging and may depend on fluctuating concentrations in urine samples [16]. At elevated exposure levels, Mn - an essential nutrient - has also been linked to neurotoxicity [8,17]. Studies have reported negative correlations between levels of Mn present in the hair of children aged 6 to 13 years and their full-scale and verbal IQ scores [8,18].

This study aimed to investigate the associations between airborne levels of Pb, manganese, Cd, chromium, and As and the intellectual function of school-age children in Makkah, Kingdom of Saudi Arabia.

## Materials and methods

Study design and sample selection

Our cross-sectional study aimed to examine the association between exposure to multiple trace elements in ambient air and IQ scores in children around Makkah. We randomly enrolled 500 children, and the participants’ parents provided their approval and signed consent forms to participate in the study. All study objectives were clearly explained to the parents. We excluded children younger than seven or older than 10 to get a consistent and reliable range of cognitive abilities within the same age group, resulting in a final sample of 430 children. Skilled doctors administered a structured questionnaire to gather demographic and socioeconomic data, including age, parents’ occupation, smoking, and drug use during pregnancy. We categorized the education levels of the children’s mothers as primary or below high school and university level or above. The Ethics Review Board for Human Studies at the Faculty of Medicine, Umm Al-Qura University, approved the study protocol per the Saudi National Committee for Bioethics (HABO-02-K-012).

Sampling of trace elements in ambient air

We used a mini volume sampler (MiniVol, AirMetrics, Springfield, OR, USA) to collect PM10 samples for 24 hours at five locations around Makkah, representing residential areas with small-to-medium industrial activities and traffic load. We placed the air sampler at a height of 10 m with a flow rate of 16.6 L/minute and set it on a 47-mm Teflon filter for 24 hours, accumulating samples weekly. We weighed and acclimated the PM10 filters at 35 to 40 °C with 60% to 70% humidity.

Trace elements analysis

We collected sample filters weekly for individual analysis. We extracted particles from each filter using nitric acid (7 mL) and ultrapure water (2 mL; American Society for Testing and Materials Type 1 Water from Millipore filtration system, MilliporeSigma, Burlington, MA, USA). We then subjected the samples to microwave-assisted acid digestion using 5 mL of concentrated nitric acid, 3.0 mL of concentrated hydrofluoric acid, 2.0 mL of concentrated hydrochloric acid, and 1.0 mL of hydrogen peroxide in each sample vessel. After sealing the sample vessels and placing them in a rotor (8×100) for microwaving, we used inductively coupled plasma-mass spectrometry with Perkin Elmer 7300 (Perkin Elmer, Waltham, MA), following the manufacturer's instructions, to measure the concentrations of Pb, Mn, Cd, Cr, and As in triplicate from the samples. The detection of trace elements was carried out by adhering to the appropriate wavelengths outlined in the United States Environmental Protection Agency Method 200.7 [19]. We compensated for analysis interferences by adjusting the processing parameters, which included modifying the background correction points (Table 1).

**Table 1:** Instrumental and data acquisition parameters of the ICP-Perkin Elmer 7300 (Perkin Elmer, Waltham, MA, USA). ICP, inductively coupled plasma; RF, radio frequency

Instrumental parameters		Data acquisition	
RF power	1400 W	Measuring mode	Segmented scan
Argon gas flow	13-16 L/minute	Point per peak	5
Nebulizer	1.0 L/minute	Scans/replicates	6
Plasma	18.0 L/minute	Replicate/sample	6
Sample uptake rate	190 s	Integration time	398.6 s

Quality assurance and quality control procedures

To ensure the reliability of our findings, we implemented a quality assurance and quality control (QA/QC) procedures for each sample by analyzing a control sample alongside the test samples. We verified the reproducibility and linearity of each analysis and created a linear calibration curve with blank and five-point calibration curves, which included concentrations of 0.01, 0.1, 0.2, 0.5, and 1.0 ppm for each of the five element standards. All samples containing measured concentrations of elements, including the QC samples, fell within the calibration curve's range. We utilized 2% nitric acid to analyze all calibration, QC, and production samples. For all 0.001-ppm QC checks, the determined concentration was within 20% of the true value, and the relative standard deviation (SD) was less than 6%.

Measurement of children’s IQ score

We used Raven’s Standard Progressive Matrices (SPM) to assess children’s intelligence [20]. Composed of 60 questions across five groups, Raven's SPM assesses a person's discrimination, similarity comparison, comparative reasoning, serial relation, and abstract reasoning abilities [8]. The difficulty level increases gradually within each group from the first item, and the maximum obtainable score is 60. We administered the tests to each child to be completed within 40 minutes. Before delivering the test, investigators explained the test requirements to parents and their children. We used age-adjusted *z* scores to estimate children’s intelligence for ease of analysis. The formula for determining the *z* score was as follows: *z* score = (IQ - ‾IQ)/SIQ, where ‾IQ represents the average intelligence of children of a certain age and SIQ denotes the SD for children of a certain age [8].

Statistical analysis

In our study, we conducted a descriptive analysis of the demographic characteristics of the participants. Continuous variables with concentrated and discrete trends were characterized using means ± SDs. Furthermore, we calculated Spearman correlation coefficients to examine the relationships among the levels of the five heavy metals. Using linear regression models, we evaluated the associations between the levels of each metal and children's intelligence, treating them as separate predictors [8]. We also utilized multiple metal linear regression models to estimate the relationship between co-exposure to the five-metal mixture and children's intelligence. We adjusted all models for covariates such as gender, maternal education levels, and smoking status during pregnancy while analyzing children's intelligence at baseline [8]. We processed the data using IBM SPSS Statistics for Windows, Version 21.0. (IBM Corp., Armonk, NY, USA). Furthermore, all statistical tests were two-sided, with a significance level of *P *< 0.05.

To evaluate the combined effect of heavy metals on continuous outcomes, we employed Bayesian Kernel Machine Regression (BKMR), designed to achieve several objectives such as detecting and estimating the overall mixture effect, identifying the pollutant or group of pollutants responsible for observed mixture effects, visualizing the exposure-response function, and detecting interactions among individual pollutants. The main concept of BKMR is to model exposure using a kernel function [21]. Specifically, the general modeling framework is:

Yi = h (Pb, Mn, Sb, Sn, Ti) + βXi + εi.

In our analysis, the response for each child (*I* = 1,…,*n*) was represented by Yi, while the exposure-response function among the five heavy metals was denoted by *h*, and Xi and β were used to indicate covariates and their respective coefficients [8]. To carry out our analysis, we utilized the BKMR technique, which integrates Bayesian and statistical learning methods to iteratively regress an exposure-response function using a Gaussian kernel function. Additionally, we assessed the posterior inclusion probability for each heavy metal to measure its variable importance. Values closer to 1 indicated greater importance, while values closer to 0 indicated less significance. To evaluate the univariate exposure-response curves among the metals, we estimated the predicted IQ scores for each level of the metal of interest while maintaining all other metal exposures at the median, 25th percentile, or 75th percentile [8].

## Results

Study participants’ demographic characteristics

We interviewed 500 children without neurological disorders in the Makkah region. A total of 430 children were included in the study. Table 2 presents the characteristics of the study participants. The enrolled children had a mean age of 7.57 (SD 0.83) years, with 50.6% being girls.

**Table 2:** Summary data for study population. SD, standard deviation

Studied participants	Total (*n *= 430)	*P*-value
	Mean ± SD	
Age (years)	7.38 ± 0.89	<0.001
Gender		
Boys	212 (49.4%)	>0.05
Girls	218 (50.6%)	
Body mass index (kg/m^2^)	15.94 ± 2.40	>0.05
Mother education level		
Primary level	101 (23.7%)	>0.05
High school	272 (63.3%)	
University level	57 (13%)	
Smoking during pregnancy		
No	414 (96.5%)	<0.001
Yes	16 (3.5%)	
Residence at the same location		
≤5 years ago	357 (83%)	>0.05
>5 years	73 (17%)	
Maximum time that child spend outdoor		
≤8 hours per day	366 (85%)	<0.001
>8 hours per day	64 (15%)	

Levels of trace elements in ambient air

**Figure 1:**
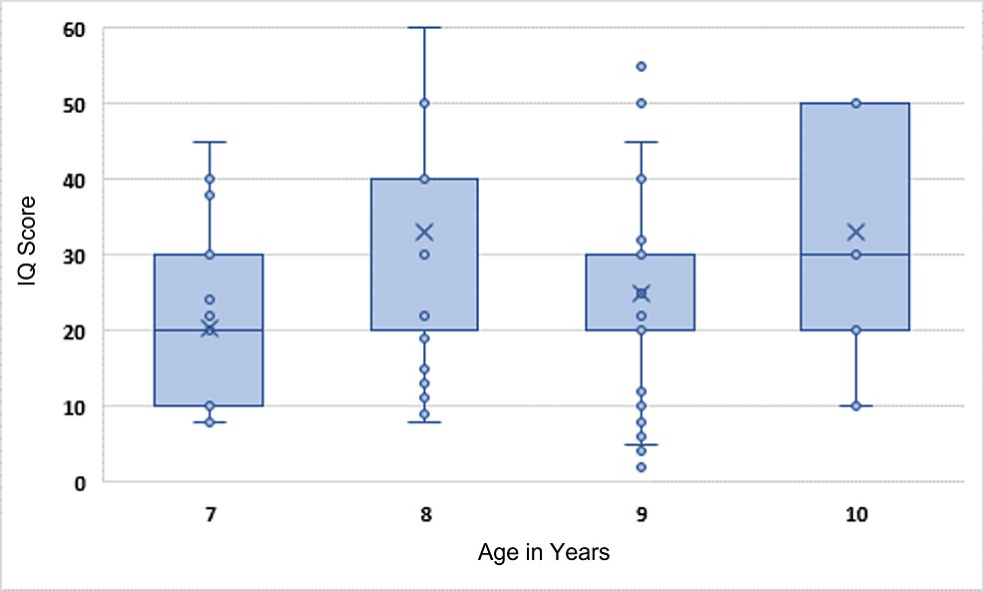
Distribution of IQ score by children’s age. IQ, intelligence quotient

**Figure 2:**
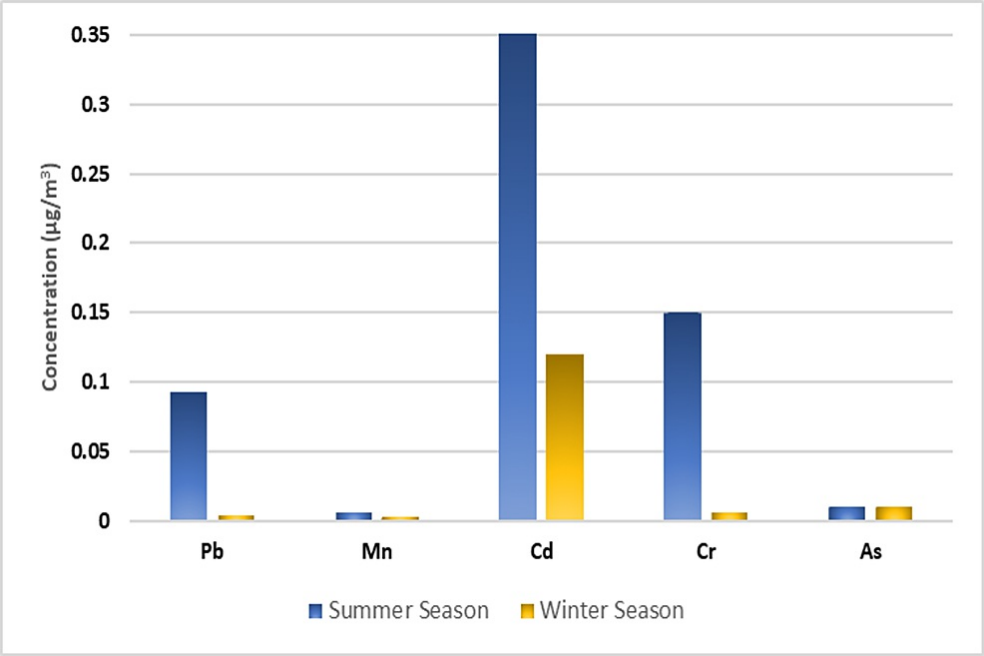
Seasonal mean concentrations of measured trace elements in PM10 at different monitoring sites. Pb, lead; Mn, manganese; Cd, cadmium; Cr, chromium; As, arsenic

Figure 1 displays the distribution of IQ by age at baseline. The mean atmospheric concentrations of Pb, Mn, Cd, Cr, and As in summer were 0.093, 0.006, 0.36, 0.15, and 0.017 µg/m^3^, respectively, while the corresponding values in winter were 0.004, 0.003, 0.12, 0.006, and 0.01 µg/m^3^, respectively (Figure 2).

Correlation of children’s IQs and combined metal exposure

To evaluate the combined effect of heavy metals on continuous outcomes, we employed the BKMR model. We found a significantly negative association between the metal mixture and IQ scores when all heavy metals were above their 50th percentiles, as compared to when all metals were at their 50th percentiles (*P *< 0.05) with the correlation coefficient (*r*) values ranging from 0.07 to 0.58. While no statistically significant difference was observed in the baseline IQ when all metal concentrations were below their 50th percentiles (*r*-value of 0.55-0.57), a significantly positive association was noted when the metal concentrations were below their 50th percentiles with (*r*) values of all combined metals Pb, Mn, Cd, Cr, and As (0.55, 0.53, 0.54, and 0.53, respectively; *P *< 0.05). Furthermore, when all metals were below their 70th percentiles, a statistically significant overall association was detected compared to when all metals were at their median value (*P *< 0.05).

## Discussion

Across all monitoring sites surrounding Makkah, the average PM10 concentration during summer was 1.5 times higher than that during winter. This seasonal increase in particulate concentration may be attributable to a combination of factors such as dust re-suspension from roads, natural dust storms, and increased automobile traffic. During the pilgrimage period in summer, PM10 concentrations in the Arafat area further increased due to heightened pollution problems from transportation, potentially contributing an unspecified amount of trace elements to air pollution. Additionally, there were significant differences in the mean concentrations of airborne trace elements between the seasons [22].

The arsenic concentration was the highest in all studied months across different seasons, consistent with another study conducted in Makkah that reported the highest concentrations of arsenic in various PM10 samples [22]. The heightened heavy metal concentration observed during summer may be attributed to high-temperature inversion. This weather phenomenon increases the number of PM10 particles near the surface due to enhanced atmospheric turbulence and blowing dust, ultimately leading to the transportation and dissemination of metal contaminants in the surrounding regions.

Our study identified independent associations between co-exposure to five metals (Pb, Mn, Cd, Cr, and As) and IQ scores in children. The BKMR models demonstrated the overall effects of the mixture. There was a significant decrease in the positive correlation as the level of multimetal exposure increased. The univariate exposure-response function revealed a negative relationship between IQ scores and each of the five heavy metals, namely Pb, Mn, Cd, Cr, and As. Specifically, our study discovered a significant negative association between Pb and children's IQ in the single-metal linear regression analysis.

Additionally, girls were found to have higher IQ levels than boys (*P *< 0.05), aligning with the findings of an earlier study in Taiwan [23]. However, another study in an industrial area in China found no significant association between urinary Pb and children’s IQ [3,8]. A potential cohort study examining sex-specific associations of neonatal and childhood exposure to eight trace elements with the cognitive abilities of 296 school-age children reported similar results [24]. Multiple studies have indicated that elevated levels of Pb in the blood can impact the intelligence of children [8,25,26]. In multiple metal linear regression models, the negative correlation between urinary lead and children's intelligence was found to be non-significant in studies in Spain (urinary Pb median = 1.04 µg/L) [27] and Italy (urinary Pb median = 1.17 µg/L) [8,28]. The same study revealed a significant association between urinary Mn and IQ scores [28]. The immaturity of the blood-brain barrier in children can lead to the accumulation of Mn in their nervous systems, which has the potential to impact the development of intelligence [8,29]. Previous studies have demonstrated an inverted U-shaped dose-response relationship between children's intelligence and Mn exposure [8].

Our study aimed to examine the evidence of relationships between multi-element exposure and children’s intelligence, using BKMR models that closely represent the actual exposure situation. Nevertheless, our study has several limitations. First, we could not follow up on metal exposure after an extended period. Second, we relied on ambient air measurements of heavy metal concentrations instead of detecting metal levels in children’s biological samples, as obtaining parental approval was challenging. Ambient air measurements may not accurately represent individual-level exposure, which can vary due to time spent indoors or outdoors, proximity to emission sources, and individual behavior. Also, our study was conducted in the Makkah region, limiting the generalizability of the results to other regions or countries with different environmental conditions, levels of heavy metal exposure, or sociodemographic factors.

Additionally, we did not consider several potential confounding factors, such as parents’ IQ, lifestyle, children’s ethnicity, or other unmeasured environmental, social, or genetic factors that could impact children’s cognitive development and potentially confound the observed associations between heavy metal exposure and IQ scores. Another limitation is that our study relied on a single measure of intelligence (IQ scores), which may not capture the full range of cognitive abilities or developmental outcomes that could be affected by heavy metal exposure. Finally, the cross-sectional design did not allow for determining causality or the temporal order of heavy metal exposure and cognitive development. A longitudinal study design would provide more robust evidence for causal relationships.

## Conclusions

According to our study findings, there is a positive correlation between children's IQ scores and co-exposure to five trace elements, namely, Pb, Mn, Cd, Cr, and As. Continuous monitoring of trace elements in children’s biological samples may be essential for understanding their impact on cognitive development. These findings highlight the importance of effective assessment and intervention strategies to mitigate the potential public health risks associated with heavy metal exposure. While our study provides insights into the association between multi-element exposure and children’s intelligence, future research should use longitudinal designs, incorporate individual-level exposure assessment, examine a broader range of cognitive abilities, and account for additional confounding factors to enhance our understanding of the impacts of heavy metal exposure on cognitive development.
